# Amyloidogenic cross-seeding of Tau protein: Transient emergence of structural variants of fibrils

**DOI:** 10.1371/journal.pone.0201182

**Published:** 2018-07-19

**Authors:** Bartosz Nizynski, Hanna Nieznanska, Robert Dec, Solomiia Boyko, Wojciech Dzwolak, Krzysztof Nieznanski

**Affiliations:** 1 College of Inter-Faculty Individual Studies in Mathematics and Natural Sciences, University of Warsaw, Banacha 2C, Warsaw, Poland; 2 Faculty of Chemistry, Biological and Chemical Research Centre, University of Warsaw, Warsaw, Poland; 3 Department of Biochemistry, Nencki Institute of Experimental Biology of Polish Academy of Sciences, Warsaw, Poland; Rijksuniversiteit Groningen, NETHERLANDS

## Abstract

Amyloid aggregates of Tau protein have been implicated in etiology of many neurodegenerative disorders including Alzheimer's disease (AD). When amyloid growth is induced by seeding with preformed fibrils assembled from the same protein, structural characteristics of the seed are usually imprinted in daughter generations of fibrils. This so-called conformational memory effect may be compromised when the seeding involves proteins with non-identical sequences leading to the emergence of distinct structural variants of fibrils (amyloid ‘*strains*’). Here, we investigate cross-seeding of full-length human Tau (FL Tau) with fibrils assembled from K18 and K18ΔK280 fragments of Tau in the presence of poly-L-glutamate (poly-Glu) as an enhancer of Tau aggregation. To study cross-seeding between Tau polypeptides and the role of the conformational memory effect in induction of Tau amyloid polymorphism, kinetic assays, transmission electron microscopy, infrared spectroscopy and limited proteolysis have been employed. The fastest fibrillization was observed for FL Tau monomers seeded with preformed K18 amyloid yielding daughter fibrils with unique trypsin digestion patterns. Morphological features of daughter FL Tau fibrils induced by K18 and K18ΔK280 seeds were reminiscent of the mother fibrils (i.e. straight paired fibrils and paired helical filaments (PHFs), respectively) but disappeared in the following generations which became similar to unpaired FL Tau amyloid fibrils formed *de novo*. The structural evolution observed in our study was accompanied by disappearance of the unique proteolysis profile originated from K18. Our findings may have implications for understanding molecular mechanisms of the emergence and stability of Tau amyloid strains.

## Introduction

The self-assembly of intrinsically disordered Tau protein into amyloid aggregates is a hallmark of *tauopathies* such as: frontotemporal dementia and parkinsonism linked to chromosome 17 (FTDP-17), progressive supranuclear palsy, corticobasal degeneration, and AD [1, 2]. In a way similar to amyloidogenesis of many other proteins, fibrillization of Tau occurs through nucleation-dependent polymerization [2–5] in which formation of amyloid nuclei is the rate-limiting step. Furthermore, as it is also the case of many other amyloidogenic proteins [6–8], even in the absence of sequence modifications, Tau has the capacity to form different structural variants of fibrils which can propagate upon seeding [9–11]. Such self-propagating polymorphism of amyloid fibrils bears a number of similarities to the propagation patterns of mammalian and yeast prions [12–19]. This has led to the adoption of term ‘strains’ originally used to describe distinct phenotypic variants of prion diseases (e.g. in [13]). The following studies on other amyloidogenic proteins and peptides (e.g. Aβ [7]), including non-pathogenic strictly *in vitro* models (e.g. insulin [6]), have pointed out that distinct amyloid polymorphs can self-assemble *de novo* from identical (in terms of amino acid sequence) monomers depending on physicochemical factors. Because amyloid-formation is kinetically controlled, such polymorphs could catalyze growth of daughter generations of fibrils while imprinting the original structure even under conditions that *de novo* favor formation of different fibrils (hence ‘*self-propagating polymorphism’*). The hypothesis that similar molecular mechanisms could underlie propagation of various prion types and amyloid polymorphs of other proteins has contributed to using the term ‘strains’ to describe polymorphs of amyloid fibrils also of proteins unrelated to prions [20]. The self-propagating polymorphism of amyloid fibrils is based on the principle of the conformational memory effect demanding that the mother template (amyloid seed) imprints its structure in incoming and integrating monomers with a high fidelity. For a variety of reasons, the persistence of such molecular imprinting may not hold during following rounds of self-seeding of amyloidogenic proteins [21]. A conformational transition to a different amyloid strain becomes more plausible during cross-seeding, i.e. when the primary structures of the seed and the recruited monomers are not identical preventing thereby an exact replication of the template structure by daughter fibril (e.g. [22–24]).

**Fig 1:**
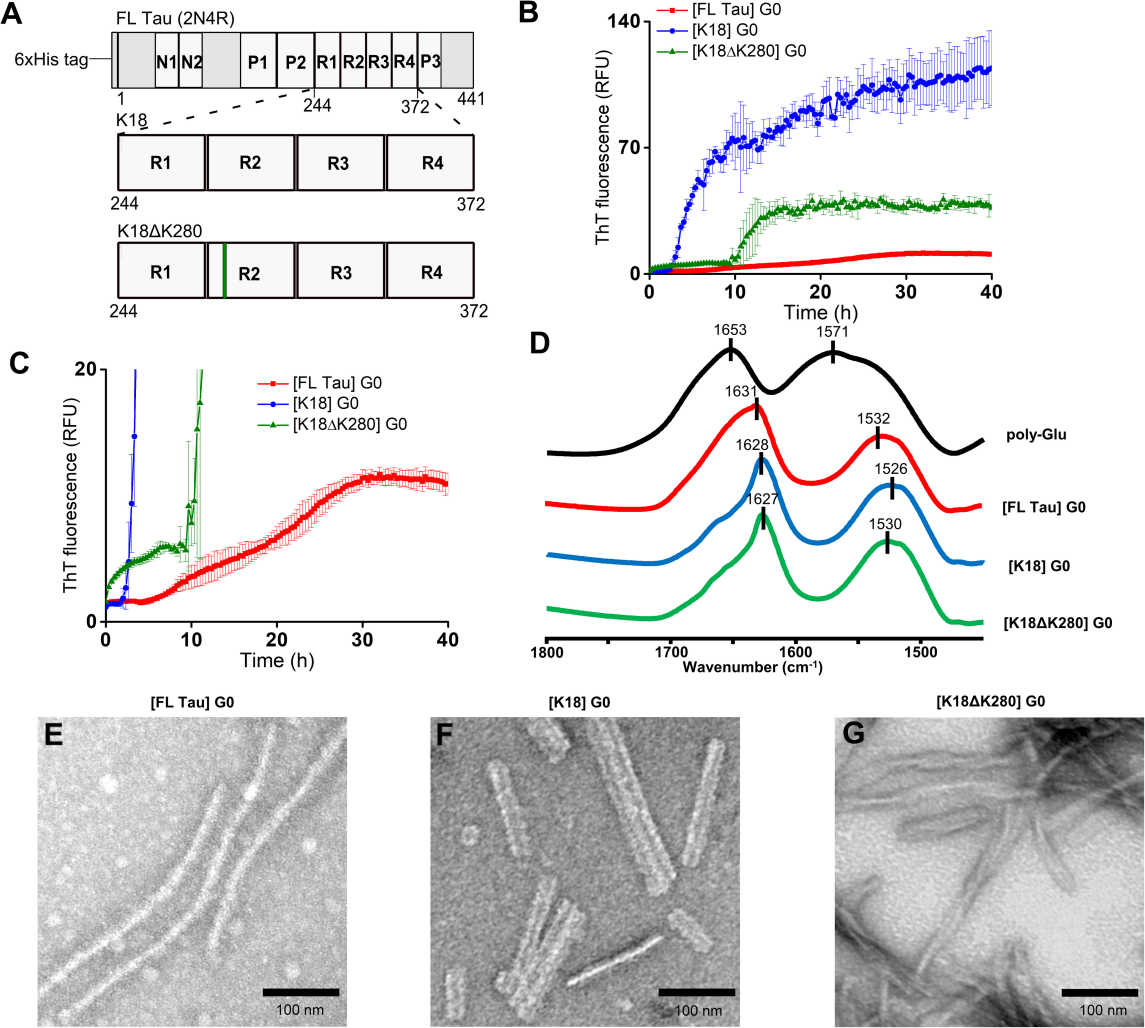
Amyloidogenesis of full-length Tau and its K18 and K18ΔK280 fragments: The mother generations (G0). (A) A schematic representation of the protein constructs used in this study: FL Tau (2N4R) containing an N-terminal His tag, K18 and K18ΔK280. The acidic inserts (N1, N2), proline-rich regions (P1, P2, P3), and microtubule-binding repeats (R1, R2, R3, R4) are indicated. (B) Kinetics of the de novo amyloid assembly of FL Tau (red), K18 (blue), and K18ΔK280 (green curve) probed by fluorescence of ThT. Error bars reflect standard deviations calculated for several trajectories measured simultaneously. (C) The intensity-magnified scale for the plot represented in B. (D) ATR-FTIR spectra of insoluble aggregates collected after 72-hour-long incubations with polyglutamate compared with the spectrum of dry film of poly-Glu in the absence of Tau polypeptides. TEM micrographs of FL Tau fibrils (E), K18 fibrils (F) and K18ΔK280 fibrils (G) formed during 72-hour-long fibrillization.

The primary goal of this study was to investigate cross-seeding of FL Tau with fibrils obtained from Tau fragments comprising the microtubule-binding repeats (MTBRs) (Fig 1A) of the wild type polypeptide (K18), and polypeptide with the pathogenic FTDP-17-linked mutation (K18ΔK280) vis-à-vis homologous self-seeding of FL Tau. These two fragments are used as highly amyloidogenic model polypeptides and may correspond to the products of *in vivo* proteolytic processing of full-length Tau which are found in tauopathy-affected brain tissues [25–27]. In this regard, truncated Tau fragments representing MTBRs are insightful polypeptide models for research on the molecular basis of Tau-related diseases. The deletion of Lys280 in Tau protein is associated with FTDP-17 [28]. The protein carrying this mutation exhibits enhanced tendency to form amyloid fibrils, much higher than other tauopathy-associated mutants [29]. The exceptionally fast aggregation was the reason we have chosen ΔK280 over other Tau mutations. Furthermore, ΔK280 mutant forms paired helical filaments resembling those found in AD [29]. Besides increased tendency to self-aggregate, ΔK280 Tau mutant shows reduced ability to promote microtubule assembly [30]. In FTDP-17, the mutation also affects the alternative splicing of exon 10 by the ablation of an exon splicing enhancer element, resulting in a decrease of 4R:3R isoforms ratio [31].

Our interests have been focused on the persistence of structural phenotypes of mother amyloid seeds from these amyloidogenic substrates upon multiple rounds of cross-seeding to FL Tau monomers in the presence of poly-Glu as a polyanionic inducer of aggregation. It is important to stress that while poly-Glu may form, under certain conditions, amyloid-like fibrils (which has been shown previously [32–34]) that requires lowering pH so that Glu side-chains are reprotonated and neutral. However, at pH 6 used in this study poly-Glu itself is unable to adopt the amyloid-like state [33]. Several biophysical and structural tools including TEM, infrared absorption spectroscopy, limited proteolysis with trypsin, and ThT fluorescence-based kinetic assays were used to track and characterize phenotypic stability of daughter generations of fibrils. In our system, FL Tau fibrillated into straight single fibrils (SFs) upon the initial *de novo* fibrillization while straight paired fibrils without periodicity and classical PHFs were assembled from K18 and K18ΔK280, respectively. Amyloid seeds obtained from these maternal generations (labelled ‘G0’) were used to induce subsequent generations of fibrils (G1, G2, and G3) through cross-seeding to FL Tau monomers. Our results demonstrate a complex interplay of molecular imprinting and conformational drift in the structural transgenerational evolution of FL Tau fibrils. Implications of our findings are discussed in the context of molecular mechanisms of Tau aggregation and their links to heterogeneous nature of tauopathies.

## Materials and methods

### Purification of Tau polypeptides

FL Tau was purified according to a modified protocol described previously [35]. Plasmid pET29b encoding human Tau (2N4R) was purchased from Addgene (Cambridge, MA, USA) and we used it as described by Hedgepeth et al. [36]. To obtain N-terminally His-tagged protein, the sequence coding Tau was recloned into pET28a vector (Novagen) using the restriction sites NdeI and XhoI. Tau protein was expressed in *E*. *coli* BL21. Bacterial cells collected by centrifugation at 11,260 x g for 15 min at 4°C were subsequently frozen at -20°C. The pellet was homogenized in extraction buffer A (4 M guanidine hydrochloride in 100 mM sodium phosphate buffer, pH 7.0, containing 19 mM 2-mercaptoethanol and 1 mM PMSF (phenylmethylsulfonyl fluoride)), sonicated and subsequently centrifuged at 142,400 x g for 35 min at 4°C. The supernatant was incubated with nickel resin (Ni-IDA XPure Agarose Resin, UBPBio) equilibrated in buffer A for 30 min. The resin was washed with 3 column volumes of buffer A, followed by wash with buffer B (20 mM imidazole, 500 mM NaCl, 19 mM 2-mercaptoethanol, 1 mM PMSF, 50 mM phosphate buffer, pH 7.0) and eluted with buffer C (500 mM imidazole, 500 mM NaCl, 19 mM 2-mercaptoethanol, 1 mM PMSF, 50 mM phosphate buffer, pH 7.0). Fractions most abundant in Tau (identified by SDS-PAGE) were boiled for 15 min and centrifuged at 142,400 x g for 35 min at 4°C. The supernatant was salted out by ammonium sulfate (at 50% saturation) and centrifuged at 142,400 g for 35 min at 4°C. The protein pellet was suspended in dialysis buffer D (80 mM NaCl, 0.5 mM PMSF, 0.5 mM DTT (dithiothreitol), 50 mM MES (2-(N-morpholino)ethanesulfonic acid), pH 6.8) and dialyzed twice against this buffer. The dialyzed preparation was centrifuged at 104,600 x g for 30 min at 4°C, and loaded on SP-Sepharose column equilibrated with buffer D. The column was washed with 3 column volumes of buffer D, followed by wash with buffer E (100 mM NaCl, 0.5 mM PMSF, 0.5 mM DTT, 50 mM MES, pH 6.8) and eluted with buffer F (500 mM NaCl, 0.5 mM PMSF, 0.5 mM DTT, 50 mM MES, pH 6.8). Fractions rich in Tau were salted out with ammonium sulfate at 50% saturation. The pellet of Tau obtained after centrifugation at 142,400 x g for 35 min at 4°C was dissolved in dialysis buffer G (50 mM NaCl, 0.5 mM PMSF, 0.5 mM DTT, 50 mM MES, pH 6.8) and dialyzed twice against this buffer. The dialysate was centrifuged at 77,100 x g for 30 min at 4°C. The supernatant containing pure Tau was aliquoted and stored at -80°C. Since His-tagged FL Tau has been used successfully in the previous studies employing polyanionic inducers (e.g., [26], [35], [49]) we have followed this approach in our work.

K18 (Gln244-Glu372, numbering based on the isoform 2N4R, additionally containing Met at the N-terminus) also known as Tau 4 repeat domain (Tau-4RD), and K18ΔK280 (K18 containing K18ΔK280 FTDP-17 mutation: a deletion of lysine at a position 280) were purified as described previously [37] with modifications. The pNG2 plasmids (derivatives of commercial vector pET3a (Novagen), containing ampicillin resistance) encoding K18 and K18Δ280 were kindly provided by Dr. J. Biernat. The cDNA sequences encoding the truncated Tau polypeptides were introduced into pNG2 between NdeI and BamHI restriction sites. The bacterial pellets were homogenized in extraction buffer H (50 mM MES, 500 mM NaCl, 1 mM MgSO_4_, 1 mM EGTA, 5 mM DTT, pH 6.8), sonicated by four cycles of sonication (30 s each, 15 s breaks), and subsequently boiled for 20 min. The preparation was cooled on ice and centrifuged for 35 min at 142,400 x g, 4°C. The supernatant was dialyzed twice against two portions of buffer I (20 mM MES, 50 mM NaCl, 1 mM MgSO_4_, 1 mM EGTA, 19 mM 2-mercaptoethanol, 0.1 mM PMSF, pH 6.8). The dialyzed sample was centrifuged at 104,600 x g, 30 min, 4°C, and loaded on the SP-Sepharose column equilibrated with buffer I. The column was washed with 3 column volumes of buffer I, followed by wash with buffer J (20 mM MES, 100 mM NaCl, 1 mM MgSO_4_,1 mM EGTA, 19 mM 2-mercaptoethanol, 0.1 mM PMSF, pH 6.8) and eluted with buffer K (20 mM MES, 500 mM NaCl, 1 mM MgSO_4_,1 mM EGTA, 19 mM 2-mercaptoethanol, 0.1 mM PMSF, pH 6.8). Fractions most abundant in K18/K18Δ280 were salted out by ammonium sulfate at 50% saturation. The pellet of pure K18/K18ΔK280 obtained after centrifugation for 35 min at 142,400 x g, 4°C, was dissolved in buffer L (20 mM MES, 50 mM NaCl, 1 mM MgSO_4_, 1 mM EGTA, 2 mM DTT, 0.1 mM PMSF, pH 6.8) and dialyzed twice against this buffer. The dialysate was centrifuged for 30 min at 77,100 x g, 4°C. The supernatant was aliquoted and stored at -80°C. The concentration of Tau polypeptides was determined by BCA protein assay. Purity of preparations was assessed using SDS-PAGE.

### Preparation of unseeded and seeded generations of Tau fibrils

To obtain the mother generation (G0) of Tau fibrils, 40 μM recombinant Tau polypeptides (FL Tau, K18, K18ΔK280) in the “assembly buffer” containing 2 mM DTT (Sigma), 1 mg/ml 3,000 Da poly-Glu (from Alamanda Polymers), 0.02% NaN_3_, 10 mM phosphate buffer, pH 6.0 in the volume of 500 μl were incubated at 37°C with continuous orbital shaking (400 rpm) for 72 hours in an Eppendorf ThermoMixer. At least, three replicates of each sample were prepared to obtain the desired amount of fibrils. Subsequently, samples were centrifuged for 30 min at 112,817 g, 25°C. Supernatants were discarded and pellets suspended in 10 mM phosphate buffer, pH 6.0 containing 0.02% NaN_3_. The procedure was repeated one more time and fibrils were suspended in appropriate volume of distilled water (50–100 μl) containing 0.02% NaN_3,_ after which the concentration of fibrils was measured by BCA protein assay. The fibrils were stored at 4°C. Amyloid seeds were obtained by sonication of 1:99 diluted fibrils at 25% of maximal power output of 125 W (5 cycles of 15 seconds sonication with 10 seconds breaks) using Q125 sonicator (QSonica). To obtain G1 generations, a portion of 1% of amyloid seeds of FL Tau, K18 or K18ΔK280 was added to the assembly buffer containing 40 μM FL Tau. Preparations were incubated under the same conditions as G0. Through the repetition of the same procedure G2 and G3 generations of daughter fibrils were obtained.

### ThT fluorescence assay

**Fig 2:**
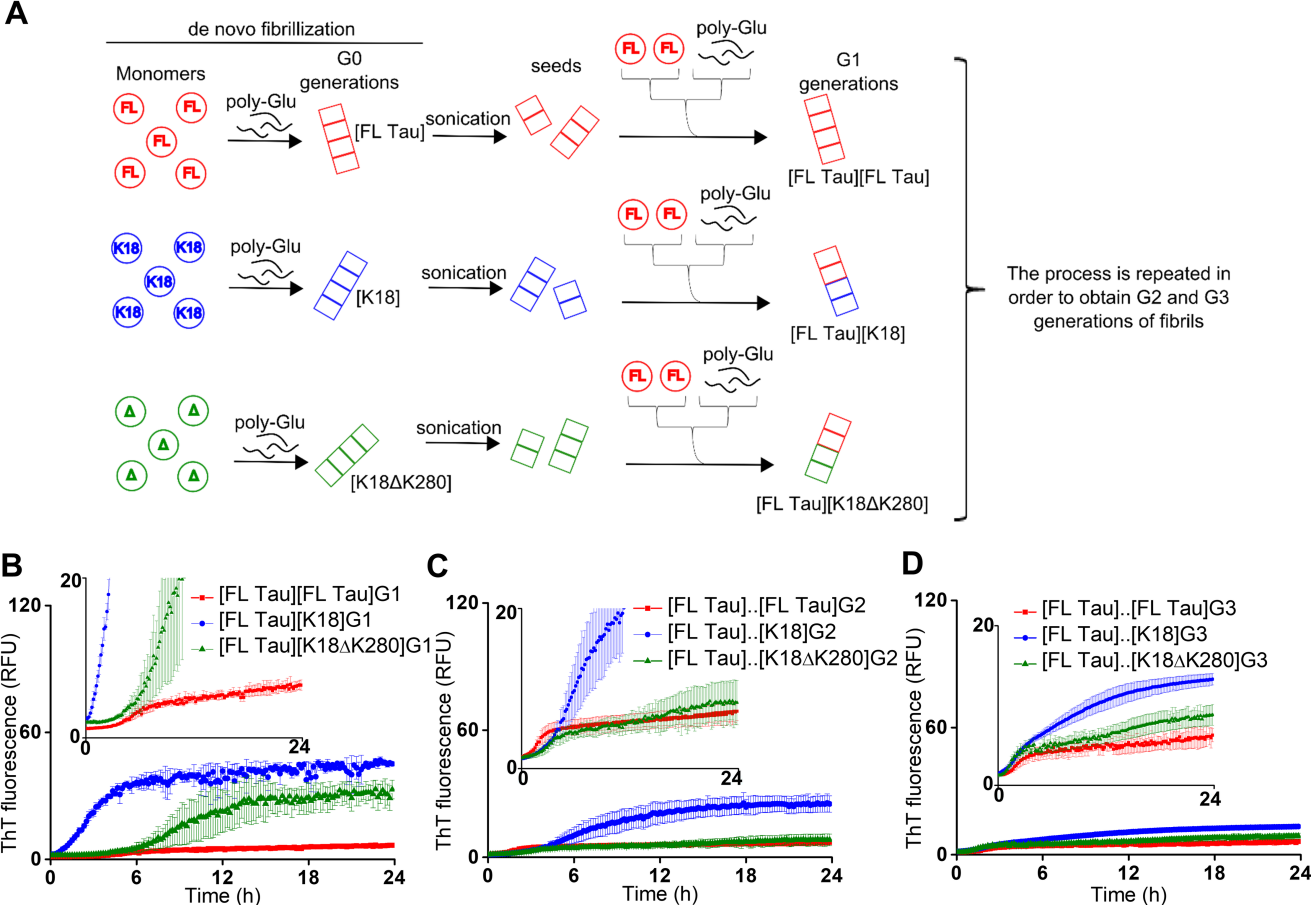
Formation of G1, G2 and G3 generations of Tau amyloid fibrils. (A) A scheme of preparation of mother (G0) *de novo* formed fibrils from monomers of FL Tau, K18 and K18ΔK280, and daughter fibril generations of FL Tau (G1, G2 and G3) obtained through seeding of FL Tau monomers with various templates. Kinetics of the self-assembly of G1 (B), G2 (C), and G3 (D) fibrils probed by ThT fluorescence. Insets show the same plots with magnified intensity scales.

A 1 mM aqueous stock solution of ThT (Sigma) was prepared and filtered through a 0.22 μm filter. Samples at the volume of 150 μl containing 20 μM Tau polypeptides, 2 mM DTT, 0.5 mg/ml 3,000 Da poly-Glu (Alamanda Polymers), 0.02% NaN_3_, 20 μM ThT, 10 mM phosphate buffer, pH 6.0 were pipetted into 96-well black plate (Greiner). Because the kinetic assays consumed relatively large quantities of costly Tau polypeptides we have decided to use them at lowered concentrations (20 μM) compared to the conditions of fibrillization in Eppendorf tubes (40 μM). Control experiments have shown that this change does not affect the key characteristics of the fibrillization kinetics (S1 Fig). In the case of seeded reactions, 1% seed (0.2 μM) was added to a well. The plate was sealed with a plastic adhesive tape (Thermo Scientific Nunc Sealing Tape) to avoid evaporation of samples. Measurements were carried out at 37°C with orbital agitation of 420 rpm. The minor difference in agitation rates applied for: (i) the in-situ monitoring of aggregation kinetics using ThT-fluorescence assay coupled to plate reader (420 rpm) vis-à-vis (ii) the conditions used for the de novo growth of mother fibrils in the Eppendorf ThermoMixer (400 rpm) arises from different sets of discrete rate values that can be selected on both accessories (i.e. the plate reader and the thermomixer) and does not itself alter the outcome of the aggregation process. Data were collected during 24, 48 or 72 hours using Fluoroskan Ascent™ Microplate Fluorometer (Thermo) equipped with a 440-nm excitation/485-nm emission filter pair. Acquisition of all kinetic data was carried out at least three times for each sample under given conditions. The error bars on kinetic plots (e.g. Figs 1B, 1C, 2B, 2C and 2D) were calculated as standard deviations for sets of three independently acquired kinetic trajectories.

### Transmission electron microscopy

**Fig 3:**
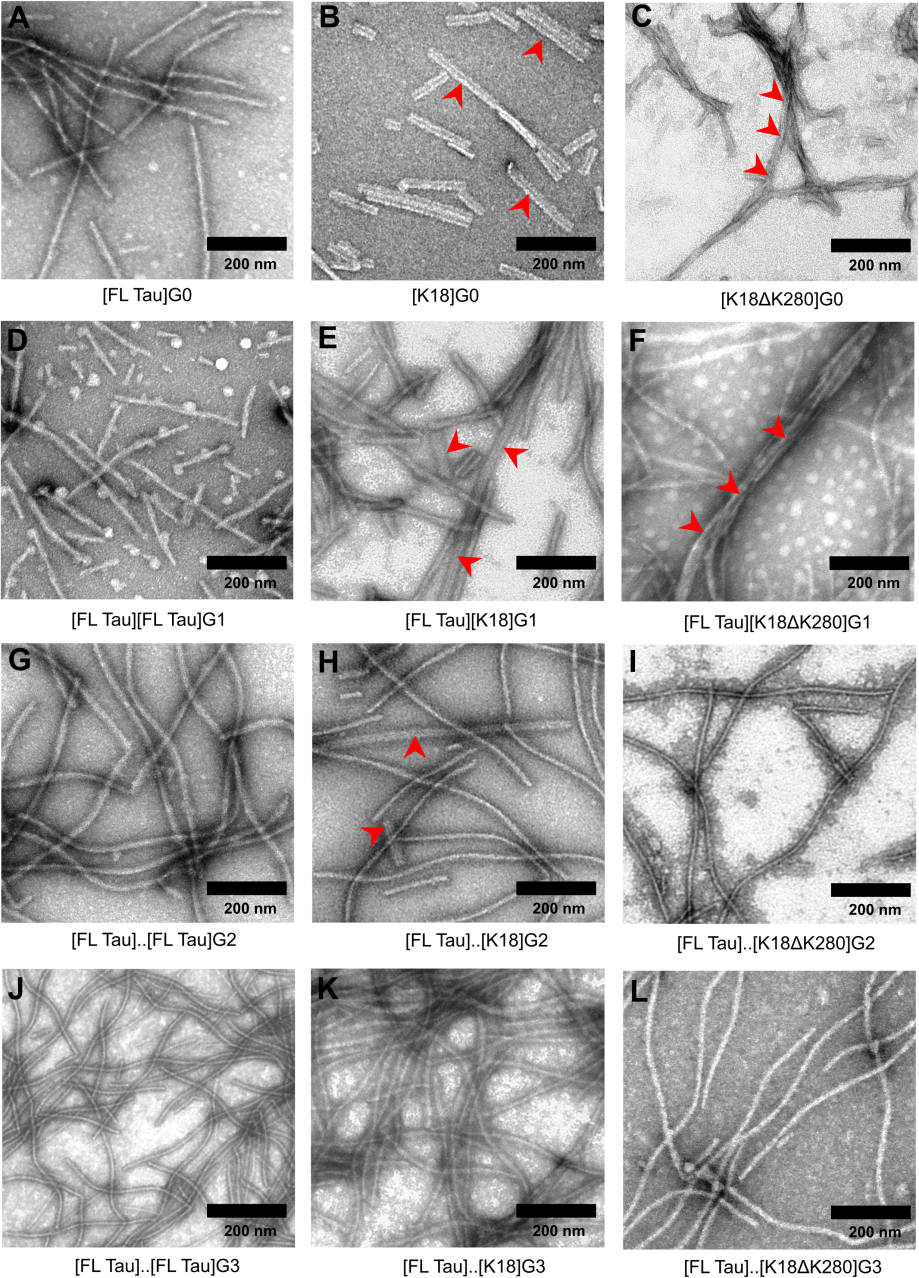
TEM images of G0, G1, G2 and G3 generations of Tau amyloid fibrils. Red arrowheads indicate morphological variations in the fibrils: paired straight structures formed by K18 and propagated on to G1 and G2 generations of FL Tau fibrils (panels B, E, H), or PHF-like structures formed by K18ΔK280 and propagated on to FL Tau fibrils (panels C, F). Representative images were chosen. Scale bar of 200 nm applies to all micrographs.

Pellets of amyloid fibrils obtained as described above were re-suspended in 10 mM phosphate buffer, pH 6.0 to a concentration of 400 μg/ml. Ten microliters of these samples were applied to a 400-mesh cooper grids (TedPella, USA) for 40 s. Subsequently, negative staining with 2% (w/v) aqueous solution of uranyl acetate (SPI Supplies, West Chester, PA, USA) was performed. The micrographs were collected by means of High Performance Transmission electron microscope JEM 1400 (JEOL Co., Japan, 2008) equipped with 11 Megapixel TEM Camera MORADA G2 (EMSIS GmbH, Germany). All TEM images used for the preparation of Figs 1 and 3 were carefully selected (from at least three experiments) to be representative for given fibrillar specimen.

### Limited proteolysis

Limited proteolysis by trypsin: a 20 μg/ml trypsin (‘TPCK treated’ from Sigma) solution was prepared in 1 mM HCl. Fibrils from generations G1, G2 and G3 at 917 μg/ml (corresponding to 20 μM FL Tau) concentrations were digested with trypsin at a concentration of 1.83 μg/ml (Tau: trypsin mass ratio was 500:1) in 20 mM phosphate buffer, pH 7.4. The samples were incubated at 37°C for 24 hours in the Eppendorf ThermoMixer. To quench the reaction, PMSF was added to a final concentration of 2 mM. Samples were boiled for 3 min in the presence of SDS-PAGE sample loading buffer and digestion products were analyzed using 15% polyacrylamide gel electrophoresis under reducing conditions according to the method of Laemmli [38].

**Fig 4:**
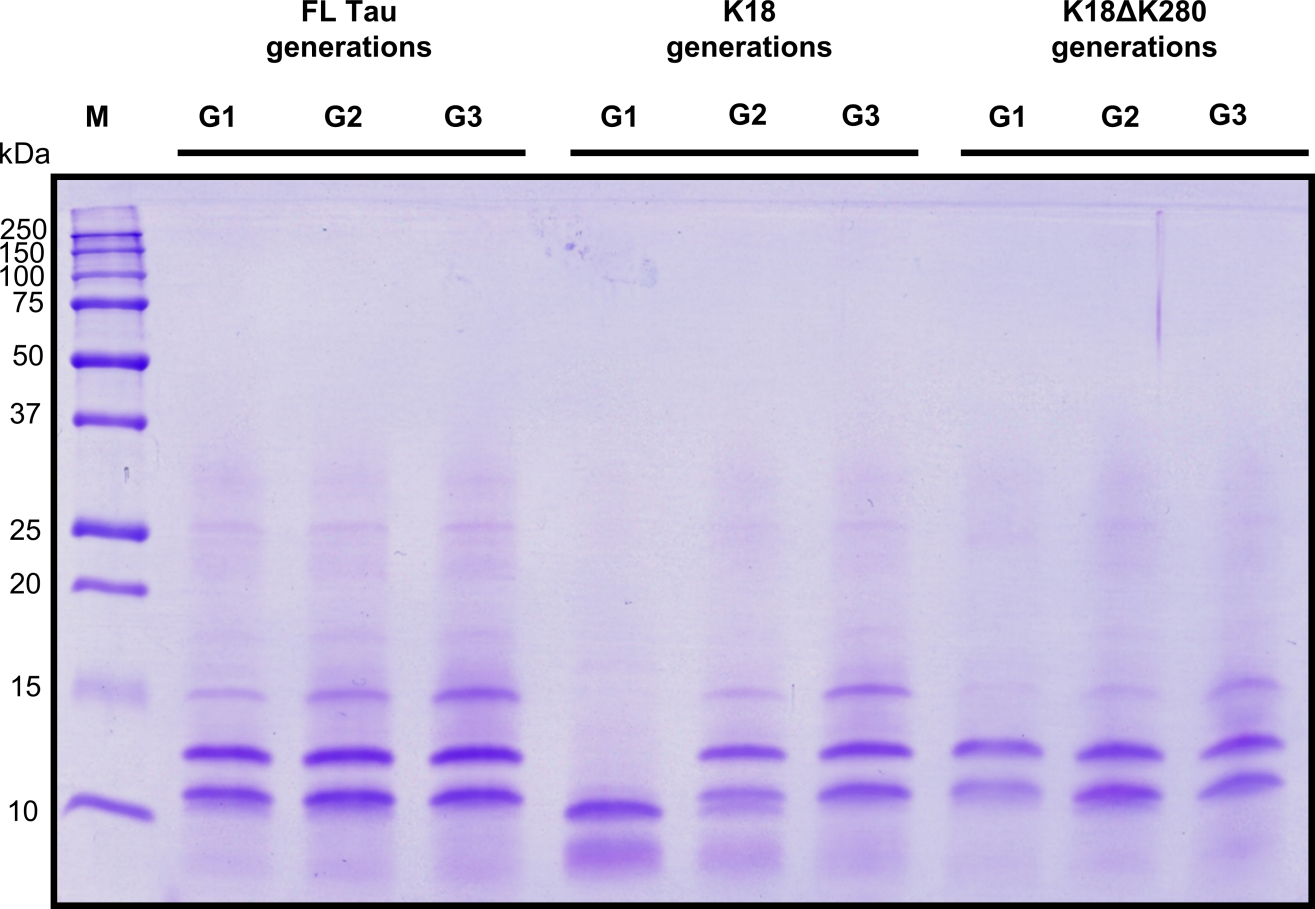
Limited proteolysis of G1, G2 and G3 generations of Tau fibrils by trypsin. SDS-PAGE analysis showing proteolytic patterns of enzymatically digested fibrils (formed during 72-h fibrillization). The lanes correspond to products of trypsin-digested G1..G3 daughter fibrils of FL Tau induced by homologous seeding (FL Tau generations) and cross-seeding (K18 / K18ΔK280 generations). M lane shows molecular weight marker.

Limited proteolysis by Proteinase K: a 35 μM Proteinase K (Proteinase K from *Tritirachium album*, Sigma) solution was prepared in 1 mM CaCl_2_ and subsequently diluted to a 350 nM concentration. G1 generations of fibrils at 917 μg/ml concentrations were digested with Proteinase K at a concentration of 70 nM in 20 mM phosphate buffer, pH 7.4 supplemented with 1 mM CaCl_2_. The samples were incubated at 37°C for different times (10, 20, 30 and 60 min) in the Eppendorf ThermoMixer. To quench the reaction, PMSF was added to a final concentration of 2 mM. Samples were boiled for 3 min in the presence of SDS-PAGE sample loading buffer and digestion products were analyzed using 15% polyacrylamide gel electrophoresis under reducing conditions according to the method of Laemmli [38]. The SDS-PAGE gel images used for the preparation of Fig 4 and S4 Fig were carefully selected (from at least three experiments) to be representative for given type of sample.

### ATR-FTIR (attenuated total reflectance Fourier transform infrared) spectroscopy

6-μl sample portions of fibrils from G0, G1, G2 and G3 generations, suspended in H_2_O, were applied onto single-reflection diamond ATR accessory coupled to Nicolet iS50 FTIR spectrometer (Thermo). After drying samples in air, infrared spectra were measured (for each spectrum 32 interferograms of 2 cm^-1^ nominal resolution were collected). The spectra acquisition was repeated several times (typically 3 times for given type of sample) in order to ensure its reproducibility. The spectral data processing performed with GRAMS software (Thermo) was confined to a two-point baseline correction and intensity normalization at the amide I band maximum.

## Results and discussion

The three different amyloid precursors used in this study (i.e. FL Tau, K18, and K18ΔK280) (Fig 1A) are disordered at physiological pH primarily due to excessive positive electric charge densities rendering conformations of FL Tau and its K18 fragments highly fluctuating and incapable of collapsing into a structurally defined and tightly-packed entity [39–40]. Amyloidogenesis of Tau protein requires the presence of polyanions such as heparin to compensate repulsive electrostatic interactions within and between monomers of FL Tau or its fragments [41–43]. Poly-Glu is an attractive alternative to heparin in this regard, and as such was used to trigger Tau aggregation in several previous *in vitro* studies [44–45]. Choice of poly-Glu as the polyanionic inducer of Tau fibrillization has the potential to provide insights into certain opaque issues surrounding dynamics of aggregating Tau which cannot be easily addressed using heparin; for example, the role of chirality and chiral transfer between the inducer and Tau in determining a fibrillization pattern (reviewed in [2]). Studies of Tau aggregation promoted by poly-Glu may have important biological implications. Clusters of glutamate residues in the tubulin molecule are involved in the electrostatic interactions between tubulin and microtubule-associated proteins [46]. The intrinsically disordered C-terminal tail of tubulin is rich in glutamate residues. This negatively charged tail serves as a binding site for the MTBRs of Tau [47]. Furthermore, the tubulin tail is a subject of polyglutamylation which can enhance interaction with Tau [46]. On the other hand, it has been found that microtubule hyperglutamylation may be directly associated with neurodegeneration [48].

Low molecular weight (3 kDa) fractions of poly-Glu were used to enhance aggregation of FL Tau, K18, and K18ΔK280. The optimal protein-to-poly-Glu molar ratio (established through the optimization procedure see–S2 Fig) was used for ThT-fluorescence-based kinetic investigation of *de novo* formation of mother amyloid seeds (Fig 1B). While all the three kinetic trajectories have sigmoidal appearances typical for nucleation-dependent polymerization reactions, we note significant differences between particular cases.

In our system, aggregation of FL Tau is accompanied by relatively small increase in ThT fluorescence, and the corresponding kinetic curve is flattened in comparison with the two amyloidogenic fragments of FL Tau. To highlight the shape of the kinetic curve corresponding to the formation of FL Tau amyloid, the red trace in Fig 1B has been magnified in Fig 1C (the kinetic curves of K18 and K18ΔK280 aggregation are shown for comparison). The effective transition of FL Tau monomers into fibrils was confirmed in ensuing steps with other methods used in this study. In fact, we have observed that ThT emission intensity corrected for actual protein concentration was significantly lower for [K18]..[FL Tau]G3 than for K18 G0 aggregates (data not shown). We also note for FL Tau long lag and elongation phases with the ThT emission reaching plateau after approximately 30 hours. Importantly, the seeded homologous fibrillizations of FL Tau (see discussion below) were very efficient, as the yield of fibrils was significantly higher than for the mother amyloid generation of FL Tau. A similar trend had been observed previously for heparin-induced Tau amyloid fibrils [49]. On the other hand, aggregation kinetics of K18 and K18ΔK280 reveal shorter lag-times followed by steeper elongation phases leading to significantly higher final ThT fluorescence signals than in the case of FL Tau. The end fluorescence plateau is well-defined for K18ΔK280, whereas for K18, after termination of the fast elongation phase (approximately 8 hours into the process), a slow increase in the emission intensity continues for at least the following 32 hours. A slowdown (or plateau) in aggregation kinetics involving an amyloidogenic protein and a co-assembling component (poly-Glu) could have trivial roots in the pool of the latter being exhausted. Therefore, we have carried out control experiments showing that a late addition of further portions of poly-Glu to aggregates at the plateau stage does not result in increase of ThT fluorescence (S3 Fig)–i.e. the observed plateaus correspond to exhaustion of–for example–free K18 monomers. As an additional control, we analyzed kinetic curves of ‘isolated’ poly-Glu in the assembly buffer (pH 6.0 in which polypeptide chain of poly-Glu remains random-like) observing no increase in ThT fluorescence (see S2 Fig). This observation proves that poly-Glu does not form amyloid aggregates in our experimental settings.

Subsequently, infrared absorption spectroscopy and electron microscopy were used to characterize mother (G0) generations of fibrils. In ATR FT-IR spectra of *de novo* formed fibrils shown in Fig 1D, the conformation-sensitive amide I band is at approximately 1628 cm^-1^ for K18 and K18ΔK280, and slightly blue-shifted (to 1631 cm^-1^) for FL Tau amyloid. This frequency range for non-deuterated protein samples points to parallel β-sheet conformation as the main secondary element of fibrils. Apart from the tiny spectral shift, a more significant difference between fibrils from full-length protein and its fragments consists in the asymmetric broadening of the amide I band observed only in the spectrum of the former. For FL Tau amyloid, it seems that the narrow spectral component attributed to β-sheets is overlapped with broad absorption around 1650 cm^-1^ originating from disordered conformations [50]. In the spectrum of pure fully ionized poly-Glu, another broad band at approximately 1570 cm^-1^ assigned to antisymmetric stretching vibrations of side chain carboxylate groups [51] is observed (partly overlapped by amide II band at ca. 1553 cm^-1^). While it is tempting to estimate amount of poly-Glu trapped within fibrils through deconvolution of the corresponding spectra, this task proved to be unfeasible due to significant bandwidth of this peak and the tendency to change frequency upon binding to cationic ligands.

The following TEM analysis revealed morphological differences in G0 generations of FL Tau, K18, and K18ΔK280 amyloid fibrils (panels E, F, and G of Fig 1, respectively) formed during 72-h-long incubation with poly-Glu. While FL Tau produced long single fibrils ~10–15 nm in diameter and with slight tendency to bend, straight paired fibrils ~25–30 nm in width (without periodicity) were observed for K18 and twisted fibrils resembling classical PHFs with ~86–96 nm periodicity predominate in the samples of K18ΔK280. As was reported previously, PHFs isolated from AD brains have twisted appearance with 80 nm periodicity [52–53]. Morphologically similar PHFs were also assembled *in vitro* from recombinant K18ΔK280 in the presence of heparin as well as in the absence of an anionic inducer [52, 54]. Untwisted fibrils of about 10 nm diameter have been described previously for FL Tau (1N4R isoform) aggregated in the presence of heparin [49]. They resemble the fibrils found in our preparations of full-length protein.

Having established conditions of poly-Glu-assisted growth of mother amyloid fibrils from FL Tau and K18/K18ΔK280 fragments we have designed the key experiments in which new portions of FL Tau monomers would be seeded in the presence of poly-Glu with sonicated pre-formed G0 fibrils from all three amyloidogenic substrates (seeds). Thus-obtained G1 generations of daughter FL Tau fibrils would be collected, characterized and, after sonication, used again as seeds to induce G2 generation of FL Tau fibrils. The protocol continued up to induction of G3 generation of fibrils, as described in the scheme in Fig 2A. ThT fluorescence was used to track kinetics of formation of the following generations of FL Tau fibrils and the corresponding plots are presented in panels B-D of Fig 2. Besides expected shortening of lag-phases observed, for instance, when G1 fibrils are induced through seeding FL Tau monomers with mother K18 templates (compare Fig 1B and Fig 2B), one striking feature of all seeded transitions is the gradual decay of ThT emission intensity at the plateau in the following generations. This tendency is especially strong for G2 granddaughter generation of K18ΔK280 amyloid for which ThT fluorescence is already as low as for homologous G2 FL Tau fibrils. In other words, phenotypes of FL Tau daughter and granddaughter fibrils descending from *de novo* formed K18 and K18ΔK280 amyloid seeds gradually acquire characteristics of the FL Tau phenotype in terms of ThT-fibril interactions. The process appears to proceed faster in the case of K18ΔK280 line of daughter fibrils.

TEM was again used to assess morphological variations in the following generations of fibrils. Images gathered in the left column of Fig 3 correspond to homologous growth (G0 to G3) of FL Tau fibrils. The single straight fibrils dominate in all the following generations of self-seeded amyloid. However, the data collected for K18 (middle column) and K18ΔK280 line (right column) suggest that daughter and granddaughter generations (in the case of K18 seeded fibrils) of cross-seeded fibrils gradually depart from the mother seeds’ morphological characteristics (i.e. regular untwisted paired fibrils for K18 and PHF-like structures for K18ΔK280) and acquire morphology of unpaired straight fibrils hallmarking FL Tau amyloid. Clearly, while the K18-like paired fibrils were still observed in cross-seeded G2 amyloid, in the K18ΔK280 line, PHF forms lasted only in the first cross-seeded generation (G1). Hence, the fast disappearance of morphological K18ΔK280 phenotype appears to parallel the kinetic data presented in panels B-D of Fig 2.

These results suggest that although the structural phenotypes of K18 and K18ΔK280 fibrils are imprinted in the first cross-seeded generations of FL Tau daughter fibrils, they remain unstable and with following passages to FL Tau monomers, the FL Tau-amyloid phenotype starts to dominate.

This straightforward interpretation suggesting a monotonic structural drift toward the phenotype “selected” during *de novo* aggregation of FL Tau is, however, challenged by the results of limited proteolysis of daughter fibrils with trypsin followed by electrophoretic analysis. As shown in Fig 4, the digestion of self-seeded FL Tau fibrils from generations G1—G3 results in the formation of two major proteolytic products (in approx. equal amounts) migrating as ~11 and ~13 kDa polypeptides. Similar proteolytic pattern is observed for FL Tau cross-seeded with K18ΔK280 fibrils, in all three digested generations. Interestingly, these two proteolytic products are not present in the samples of G1 generation of FL Tau cross-seeded with K18 fibrils. Instead, unique proteolytic fragments of relative molecular weight ~8 and ~10 kDa are formed. This suggests that [FL Tau][K18] G1 fibrils expose sites susceptible to trypsin digestion different from those exposed on the other analyzed amyloid species.

It is noteworthy that this unique digestion pattern disappears in the following generations and proteolytic products characteristic for self-seeded FL Tau appear in G2 and G3 generations of FL Tau cross-seeded with K18 fibrils. This, again, indicates that structural phenotype of K18 fibrils is unstable during following passages to FL Tau monomers.

We performed additional limited digestion of G1 samples with Proteinase K. The corresponding digestion results are shown in S4 Fig. Again, FL Tau amyloid seeded by K18 exhibits different digestion pattern from that observed for two other variants (seeded by FL Tau or ΔK280) which supports the results obtained using trypsin.

Hence, the presence of the unique proteolytic fragments should be attributed to a transient structural variant of fibrils distinct in terms of proteolytic susceptibility from both FL Tau and K18 phenotypes. Such metastable (in terms of survival in evolving pool of competing amyloid polymorphs) variant could emerge because of conformational mismatch of FL Tau monomers binding to K18 amyloid seeds. The first stage of this process, i.e. induction of novel (in terms of susceptibility to trypsin) structural variant of amyloid through cross-seeding, would resemble the *conformational switching* observed upon *in vitro* cross-seeding of different rodent prions [22]. The second stage: the ultimate transition to the “wild” FL Tau amyloid phenotype could be caused again by microscopic structural switching of energetically unfavorable [FL Tau][K18]G1 fibrils to the more energetically favorable “wild” variant. Alternatively, the transition to FL Tau phenotype could take place on the level of the whole population of growing fibrils through a kinetic selection favoring fastest spreading (through accelerated elongation, fragmentation and possibly secondary nucleation) phenotype. Such scenario requires that the rates of seed-induced fibrillization would increase in following generations of amyloid, as has been recently shown for an *in vitro* structural drift of self-seeded insulin amyloid fibrils [21].

The structural phenotype of FL Tau fibrils grown in the presence of poly-Glu has a number of distinct features in terms of TEM morphology, trypsin-digestion pattern, decreased capacity to induce ThT fluorescence and broadening of amide I vibrational band, some of which could have common molecular origins. For example, the 'fuzzy coat' consisting of less-ordered parts of FL Tau backbone postulated to cover Tau fibrils [55–57] is expected to both: contribute to the broadening of the amide I band and, possibly, through restriction of access to ThT-binding β-strand ladders, lead to a decrease in dye’s emission.

In a way similar to other amyloidogenic proteins, cross-seeded fibrillization of Tau polypeptides varying in terms of amino acid sequence can be hindered by seeding barriers. It has been demonstrated that monomeric K18 and 4R full-length Tau, can be seeded with K18 as well as K19 seeds [9]. On the other hand, fibrillization of monomeric K19 and 3R full-length Tau, can be induced by K19 but not K18 seeds. Interestingly, the cross-aggregation of K18 monomers with K19 seeds leads to the formation of K18 fibrils, which acquire conformation of K19 seeds, and the ability to template K19 monomers [9]. Plausibly, the seeding barriers may be not only due to differences in primary structure but also specific conformational incompatibilities of monomers with seeds (i.e., distinct Tau conformers possessing the same amino acid sequences can have diverse seeding activity) [58]. It has also been shown that cross-fibrillization of Tau can be influenced by the content of cysteine residues, leading to Tau fibrils of various structures [49]. Mutations in Tau can also significantly affect the seeding process. For example, seed-based fibrillization of wild-type Tau (0N4R, 383 amino acids) can be induced by R406W mutant Tau seeds, while the process does not take place (or is quite inefficient) in the presence of P301L mutant Tau seeds [59]. Surprisingly, in our study, we did not observe apparent barriers in cross-seeded fibrillizations, which implies a degree of structural compatibility between seeds of FL Tau, K18, K18ΔK280 and FL Tau monomers. Moreover, we have found that seeds of K18 and K18ΔK280 are very efficient in enhancing fibrillization of monomeric FL Tau. Notably, the importance of cross-seeding between different proteins that are involved in disease-associated processes has been shown in previous studies [60–63]. Our results support observations that cross-seeding contributes to generation of amyloid polymorphism.

It is important to emphasize, that switches in amyloid structure of Tau can be affected not only by cross-seeding. It has been found that K18 fibrils grown on to K18 seeds may gradually convert into new conformers during serial homologous seeding cycles [64]. Given the abovementioned observations, structural drift in cross-seeded fibrils should be considered as a possible factor contributing to the clinically observed heterogeneous nature of tauopathies. It is tempting to speculate that structural transitions may be ubiquitous during neurodegenerative conditions, providing heterogeneous amyloid assemblies of potentially diverse biological effects. It has been previously reported that different Tau morphologies can induce distinct pathological phenotypes. For example, so-called “Tau strains”, determined by distinct amyloid morphologies, self-propagate in cell cultures and induce distinct pathologies in transgenic mice [65]. Tau strains can cause specific intracellular changes in distinct brain regions of transgenic animals, leading to diverse neuropathological presentations [66]. It has been shown that structurally distinct aggregates of Tau induce different types of neuropathology also in non-transgenic animals [67]. Importantly, in the abovementioned report it has been demonstrated that Tau preparations derived from different human tauopathies can induce diverse neuropathologies in animals. Nonetheless, currently there is no experimental evidence that the structural evolution/drift plays a role during neurodegenerative processes in humans.

The majority of studies on Tau aggregation focused on sulphated glycosaminoglycans such as heparin to induce fibrillization [68–70]. In this work, we have described unique types of Tau fibrils and the multigenerational drift of their conformational traits, using poly-Glu as a polyanionic cofactor of aggregation. Since the type of enhancer may affect the fibrillization process, the here reported structural evolution of Tau fibrils may or may not be a general phenomenon taking place also in the presence of different polyanionic enhancers. It therefore remains to be investigated whether that is indeed the case.

## Conclusions

In summary, full-length Tau when cross-seeded in the presence of polyglutamate with amyloid seeds obtained *de novo* from its two fragments: K18 and K18ΔK280 converts into daughter fibrils whose only initial generations are controlled by the conformational memory effect. In the following rounds of seeding, the phenotypes carried by mother seeds “lose” to the phenotype that emerges spontaneously in FL Tau fibrils self-assembling *de novo*. It is likely that this structural evolution has kinetic roots. The transition route from the K18 to the FL Tau phenotype is non-monotonic and is likely to proceed through a metastable state with its own unique structural characteristics. Our experiments further support the hypothesis of the prion-like nature of Tau by emphasizing the ability of the protein to assemble into polymorphic fibrils that can transmit the biological information encoded in the structure of seeds. We also show that Tau fibrils may evolve into novel variants upon repeated seeding which also parallels with prion biology (i.e. adaptation and evolution of prion strains). We provide possible insights into the role of mutations and pathological processing of Tau protein, which may lead to the emergence of short aggregation-prone fragments that may subsequently seed full-length Tau. This may lead to formation and spreading of novel amyloid aggregates of Tau that may be prone to structural changes once seeding repeats itself. We suggest that such structural evolution of amyloid assemblies may have a role during neurodegenerative conditions.

## Supporting information

S1 Fig Comparison of *de novo* fibrillization kinetics of Tau polypeptides at 20 and 40 μM concentrations. The ThT-based fluorescence assay (carried out in the presence of poly-Glu) reveals that the dilution of protein does not result in significant perturbation of the aggregation kinetics apart from the proportional reduction of the signal intensity. The relative rates of fibrillization of the three different polypeptides are maintained, as are the fine features of the kinetic traces.
(TIF)

S2 Fig Optimal conditions for polyglutamate-induced Tau fibrillization. The comparison of aggregation kinetics of FL Tau in the presence of (A) 3 kDa poly-Glu and (B) 15 kDa poly-Glu. The assembly buffer contained 20 μM FL Tau, 2 mM DTT, 0.02% NaN_3_, 20 μM ThT, 10 mM sodium phosphate buffer, pH 6.0, and different concentrations of 3 kDa or 15 kDa poly-Glu. The most efficient aggregation of 20 μM FL Tau occurred at the concentration of 0.5 mg/ml and 0.1 mg/ml of 3 kDa poly-Glu and 15 kDa poly-Glu, respectively. 3 kDa poly-Glu was a stronger enhancer of Tau fibrillization than 15 kDa poly-Glu. In the absence of Tau, poly-Glu does not form amyloid aggregates in the assembly buffer during the aggregation period (flat kinetic curves indicated in black in panels A and B).
(TIF)

S3 Fig The effects of addition of K18 seeds/poly-Glu/H_2_O to FL Tau monomers seeded with K18 seeds. At 14 hours of aggregation (at the plateau phase), the measurement was stopped and 15 μl of (A) K18 seeds (0.2 μM) (B) poly-Glu (0.5 mg/ml) or (C) H2O were added to 20 μM FL Tau protein with 1% K18 seeds (0.2 μM) in the assembly buffer (2 mM DTT, 0.5 mg/ml 3,000 poly-Glu, 0.02% NaN3, 20 μM ThT, 10 mM sodium phosphate buffer, pH 6.0). After 20 minutes, the measurement was continued. (D) The addition of 15 μl of K18 seeds (0.2 μM) to K18 seeds (0.2 μM).
(TIF)

S4 Fig Limited proteolysis of G1 daughter generations of Tau fibrils by Proteinase K. (A) Digestion of [FL Tau][K18] G1 fibrils by Proteinase K in time (10 min–lane 2, 20 min–lane 3, 30 min–lane 4, 1 h–lane 5) was carried out to find optimal time for proteolysis. (B) 10-min digested samples of [FL Tau][FL Tau] G1 (lane 2), [FL Tau][K18] G1 (lane 3) and [FL Tau][K18ΔK280] G1 (lane 4) daughter fibrils. In A and B, lane 1 shows molecular weight marker. Note different pattern of bands in the range of 15 to 20 kDa in lane 3.
(TIF)

## Abbreviations

AD: Alzheimer’s disease
ATR-FTIR: attenuated total reflectance Fourier transform infrared spectroscopy
DTT: dithiothreitol
FL Tau: full-length Tau (isoform 2N4R)
FTDP-17: frontotemporal dementia and parkinsonism linked to chromosome 17
K18: Tau polypeptide comprising 4 repeats of the microtubule-binding domain
K18ΔK280: K18 carrying the FTDP-17 mutation
K19: Tau polypeptide comprising 3 repeats of the microtubule-binding domain
MES: 2-(N-morpholino)ethanesulfonic acid
MTBRs: microtubule-binding repeats
PHFs: paired helical filaments
PMSF: phenylmethylsulfonyl fluoride
poly-Glu: poly-L-glutamic acid
SDS-PAGE: sodium dodecyl sulfate-polyacrylamide gel electrophoresis
SFs: straight fibrils
TEM: transmission electron microscopy
ThT: Thioflavin T
