# Supplementary figures and images for: Amyloidogenic cross-seeding of Tau protein: Transient emergence of structural variants of fibrils
Source: PLoS One. 2018 Jul 19;13(7):e0201182. doi: 10.1371/journal.pone.0201182 (PMC6053212; doi:10.1371/journal.pone.0201182)

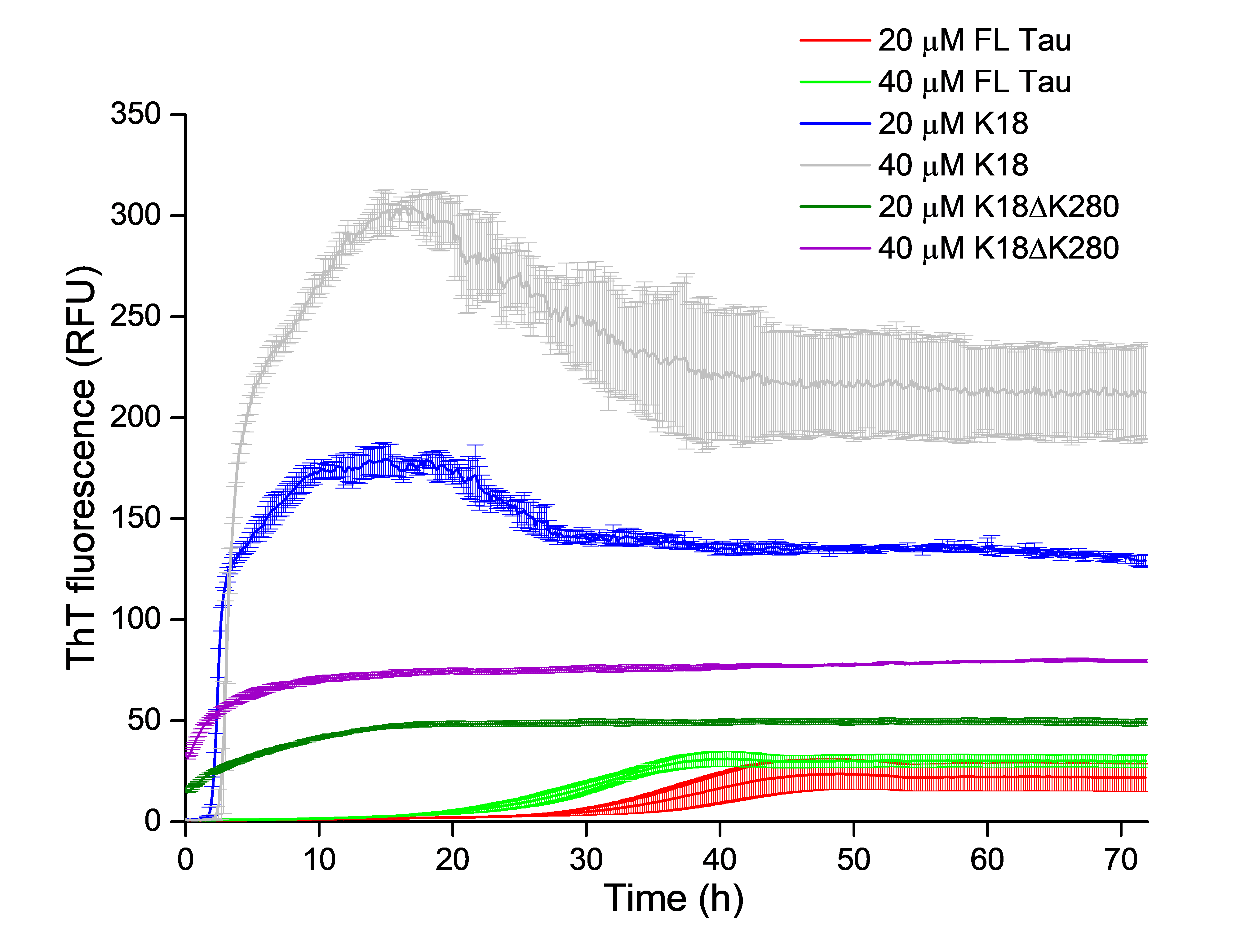

Supplement: S1 Fig — The ThT-based fluorescence assay (carried out in the presence of poly-Glu) reveals that the dilution of protein does not result in significant perturbation of the aggregation kinetics apart from the proportional reduction of the signal intensity. The relative rates of fibrillization of the three different polypeptides are maintained, as are the fine features of the kinetic traces. (TIF) [file pone.0201182.s001.tif]

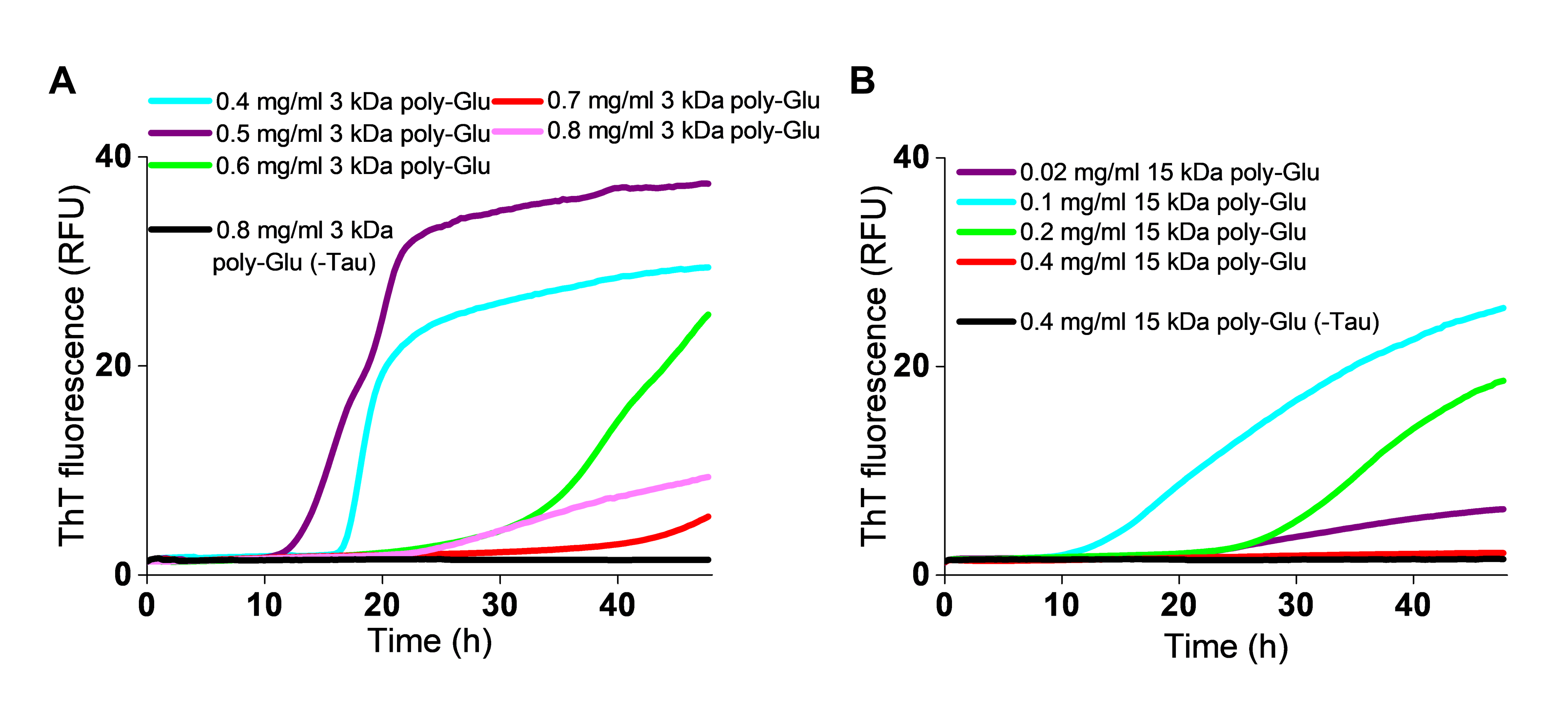

Supplement: S2 Fig — The comparison of aggregation kinetics of FL Tau in the presence of (A) 3 kDa poly-Glu and (B) 15 kDa poly-Glu. The assembly buffer contained 20 μM FL Tau, 2 mM DTT, 0.02% NaN3, 20 μM ThT, 10 mM sodium phosphate buffer, pH 6.0, and different concentrations of 3 kDa or 15 kDa poly-Glu. The most efficient aggregation of 20 μM FL Tau occurred at the concentration of 0.5 mg/ml and 0.1 mg/ml of 3 kDa poly-Glu and 15 kDa poly-Glu, respectively. 3 kDa poly-Glu was a stronger enhancer of Tau fibrillization than 15 kDa poly-Glu. In the absence of Tau, poly-Glu does not form amyloid aggregates in the assembly buffer during the aggregation period (flat kinetic curves indicated in black in panels A and B). (TIF) [file pone.0201182.s002.tif]

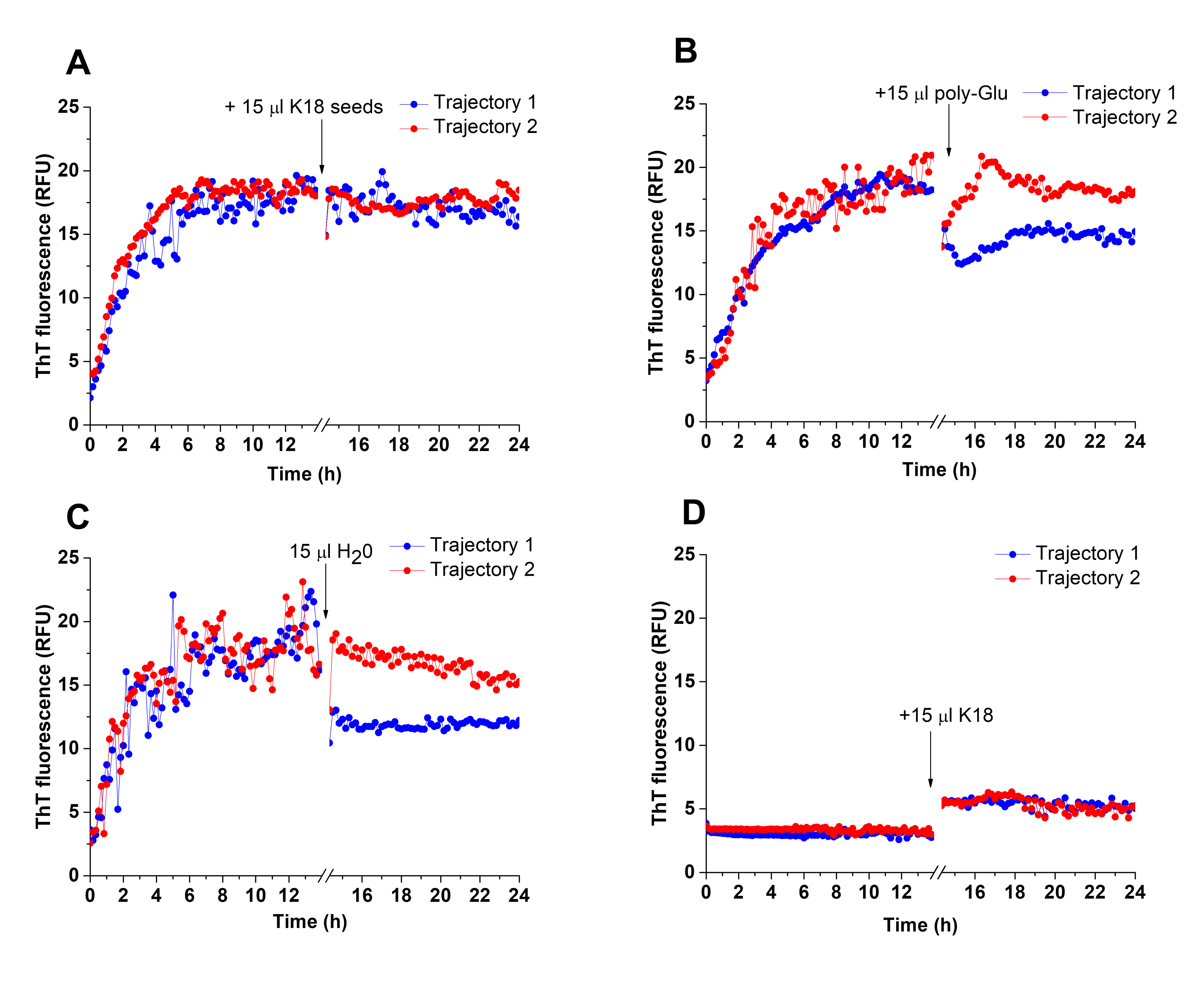

Supplement: S3 Fig — At 14 hours of aggregation (at the plateau phase), the measurement was stopped and 15 μl of (A) K18 seeds (0.2 μM) (B) poly-Glu (0.5 mg/ml) or (C) H2O were added to 20 μM FL Tau protein with 1% K18 seeds (0.2 μM) in the assembly buffer (2 mM DTT, 0.5 mg/ml 3,000 poly-Glu, 0.02% NaN3, 20 μM ThT, 10 mM sodium phosphate buffer, pH 6.0). After 20 minutes, the measurement was continued. (D) The addition of 15 μl of K18 seeds (0.2 μM) to K18 seeds (0.2 μM). (TIF) [file pone.0201182.s003.tif]

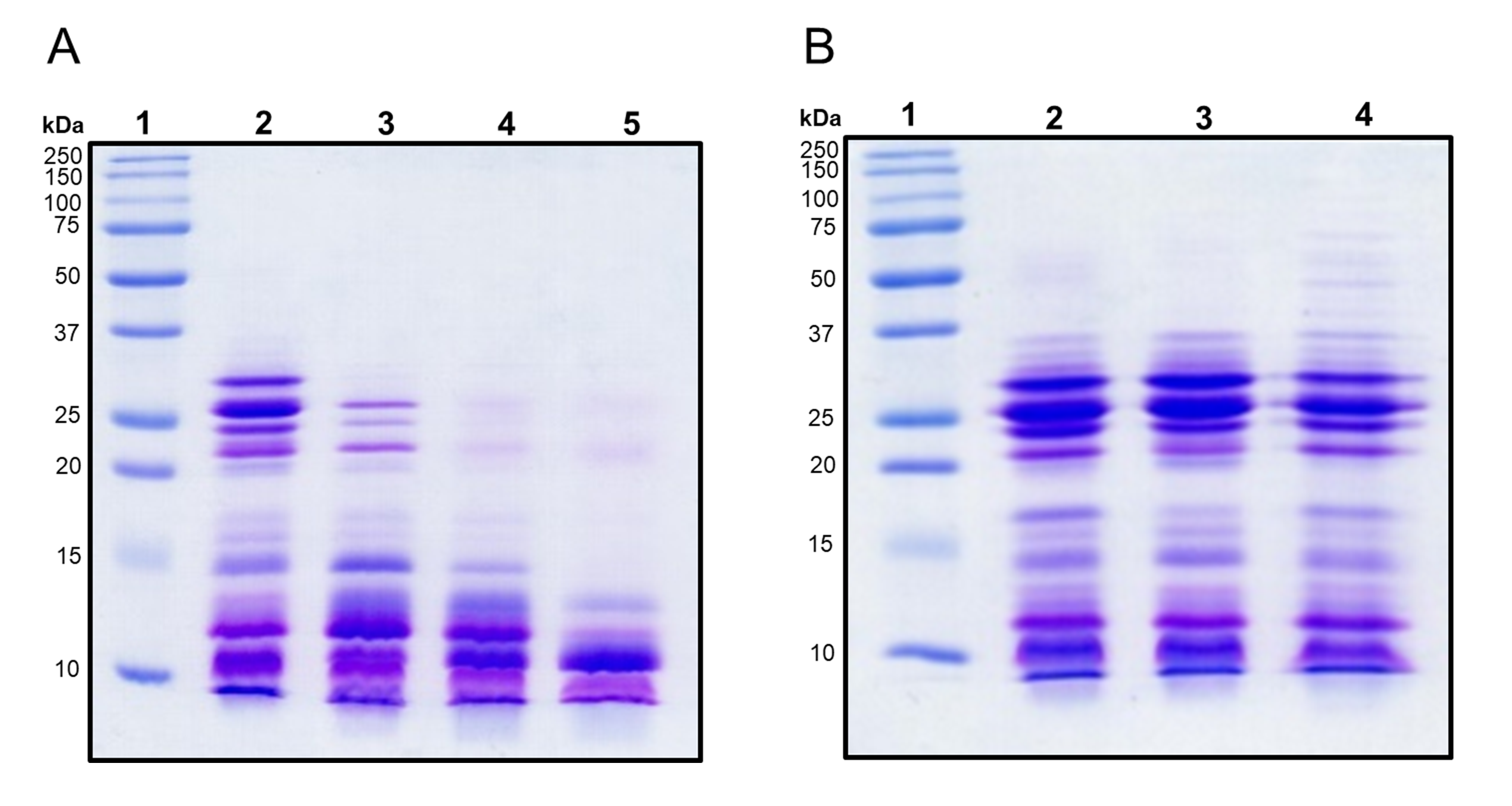

Supplement: S4 Fig — (A) Digestion of [FL Tau][K18] G1 fibrils by Proteinase K in time (10 min–lane 2, 20 min–lane 3, 30 min–lane 4, 1 h–lane 5) was carried out to find optimal time for proteolysis. (B) 10-min digested samples of [FL Tau][FL Tau] G1 (lane 2), [FL Tau][K18] G1 (lane 3) and [FL Tau][K18ΔK280] G1 (lane 4) daughter fibrils. In A and B, lane 1 shows molecular weight marker. Note different pattern of bands in the range of 15 to 20 kDa in lane 3. (TIF) [file pone.0201182.s004.tif]
